# Radial arteriovenous fistula complicated with ischemic steal syndrome after transradial cardiac catheterization: a case report and literature review

**DOI:** 10.1186/s12893-022-01562-7

**Published:** 2022-03-21

**Authors:** Fengming Gu, Jiong Yu, Jingyi Mi

**Affiliations:** 1 Medical College, Soochow University, Suzhou, Jiangsu China; 2 Department of Sports Medicine, Wuxi 9th People’s Hospital Affiliated to Soochow University, 999 Liangxi Road, Wuxi, 214062 Jiangsu China

**Keywords:** Arteriovenous fistula, Ischemic steal syndrome, Cardiac catheterization, Treatment, Case report

## Abstract

**Background:**

The radial arteriovenous fistula (AVF) is a rare complication occurring after transradial cardiac catheterization. Patients with AVF typically present with signs of venous dilation, such as swelling or palpable thrills. However, neurological complications secondary to radial AVFs are rare. This paper reported a rare case of ischemic steal syndrome that occurred 11 months after the transradial cardiac catheterization, most likely as a consequence of radial arteriovenous fistula.

**Case presentation:**

This paper described a case of a 73-year-old female, who complained of right forearm swelling and radial 1–3 fingers numbness for several months after the catheterized stent surgery through radial approach. Upon Clinical examination, this patient presented with a slight bump and palpable thrill at the distal third of right forearm, and the sensory of radial 1–3 fingers and pinch force was compromised. The Ultrasonography and computed tomography angiography (CTA) of the upper extremity revealed AVF between the right radial artery and the adjacent vein. Microsurgery was performed successfully to ligate the fistula and reconstruct the radial artery. The numbness has gradually improved about 1 week after the surgery, with no recurred swelling. The two-point pinch force and digital sensitivity recovered at the 20-month follow-up. What’s more, due to the scarcity of cases, the optimal therapy for iatrogenic radial AVF is controversial. Accordingly, we provided a literature review of previous reports of catheter-related radial AVFs and proposed an algorithm to manage them.

**Conclusions:**

We believe that once an AVF is diagnosed, early treatment options such as compression or surgery are necessary to relieve symptoms and prevent further complications. Otherwise, serious complications can occur, including the ischemic steal syndrome.

## Background

The arteriovenous fistula (AVF) is an abnormal connection between arteries and adjacent veins. It can be congenital, surgically created, or acquired via trauma or iatrogenic injury [1]. AVFs associated with cardiac catheterization are commonly reported due to percutaneous access through the femoral artery. In recent years, radial artery approaches have been gradually accepted since they dramatically reduce access site bleeding and vascular complications when compared with transfemoral access. The incidence of AVF related to transradial cardiac catheterization is only 0.04% since there are no major veins adjacent to the radial puncture site [2].

Depending on their location and etiology, AVFs can exhibit different presentations. Generally speaking, the fistula of the radial artery has fewer shunts, so it is not prone to develop clinical signs of hemodynamic significance compared with the femoral AVF [3]. Patients with AVF typically present with a palpable thrill, bruit, or pulsatile mass. There may also be an abnormal murmur over the fistula and signs of venous hypertension such as varicosities, pain, and swelling. Doppler ultrasound is the primary diagnostic tool for identifying AVFs and distinguishing them from arteriovenous malformations, hemangiomas, pseudoaneurysms, and malignant tumors [4]. CT angiography is additionally performed to accurately display the anatomical relationship between the radial artery, veins, and adjacent structures around the AVF. There are several treatment methods, such as physical compression, surgical treatment, and endovascular management, but no consensus has been reached [5].

Here, we reported a rare case of ischemic steal syndrome following radial AVF after the transradial cardiac catheterization. What’s more, we reviewed previous reports of catheter-related radial AVFs and proposed an algorithm to manage them.

## Case presentation

A 73-year-old right-handed female presented to our department with numbness in her radial 1–3 fingers and swelling in the right forearm. About 11 months ago, she was diagnosed with acute coronary syndrome and treated with catheterized stent through the radial approach. The patient felt swelling and discomfort at the puncture site 2 weeks later, and after 24 h of compression, the symptoms were slightly relieved. However, in the 6 months that followed, the swelling worsened and numbness developed in radial 1–3 fingers.

**Fig. 1:**
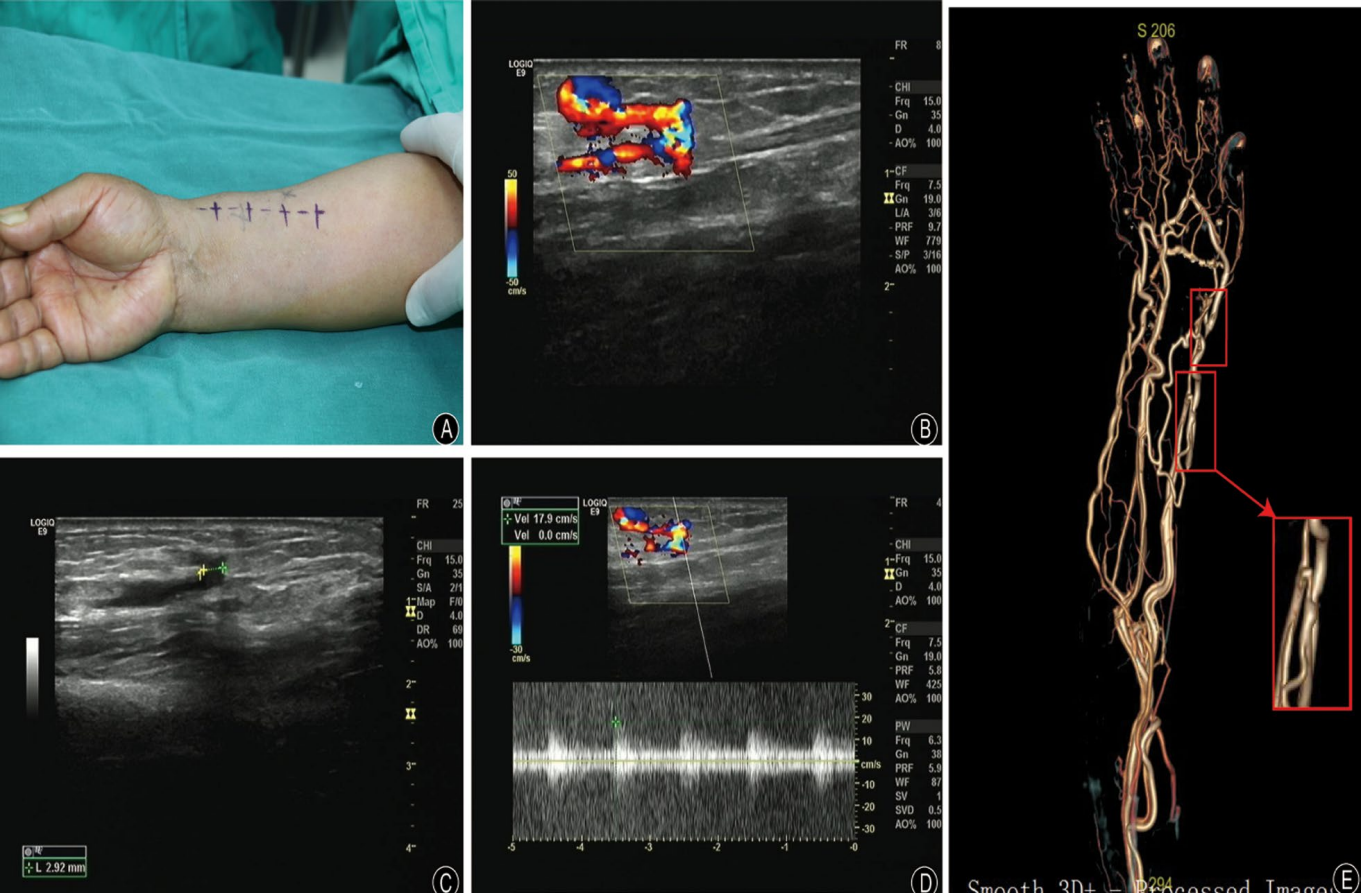
Preoperative appearance photos and images. **A** The appearance photo showed a slight bump and palpable thrill of the forearm. Doppler ultrasound showed: **B** continuous turbulent flow between radial artery and vein; **C** the diameter of the fistula neck; **D** arterial spectrum of the fistula. **E** Upper extremity CT angiography revealed AVF formation between the right radial artery and the adjacent vein around the right wrist. In addition, the continuity of the radial artery was lost just distal to the AVF on the image

The physical examination showed a slight bump and palpable thrill at the distal third of the right forearm (Fig. 1A), while Tinel’s sign and Phalen’s test (-). The pulsation of the radial artery was palpable but attenuated compared to the normal side. The capillary refill and wrist range of motion were normal. Mild atrophy was observed in the thenar muscles, the Allen test was negative, and the sensory of radial 1–3 fingers were compromised. In comparison with thumb and index finger, the middle finger has the worst sensory function. Specifically, the Static two-point discrimination and Semmes–Weinstein monofilament examinations at the middle finger were 8 mm and 4.56, respectively (NC-70142-HKP, North Coast Medical, USA). The Ultrasonography revealed a fistula between the radial artery and vein (Fig. 1B), with an inner diameter of 2.9 mm and a maximum flow velocity of 17.9 cm/s (Fig. 1C, D). Further CT angiography confirmed the presence of a fistula between the radial artery and the adjacent vein. In addition, the continuity of the radial artery was lost just distal to the AVF on the image (Fig. 1E).

**Fig. 2:**
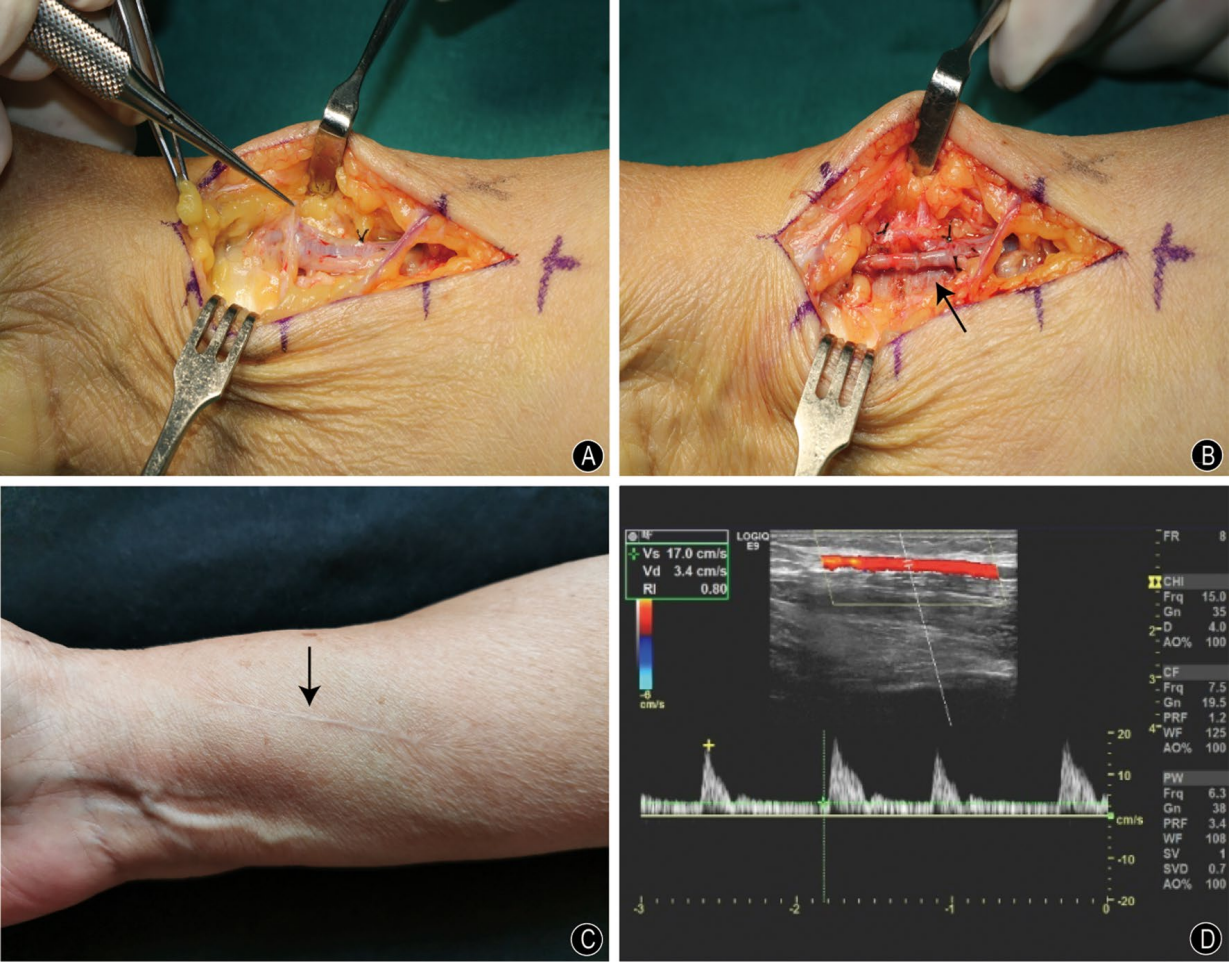
Intraoperative and postoperative pictures. **A** The artery and the vein within the fistula were mixed up. **B** After resecting and ligating the fistula, the identified radial artery was anastomosed (arrow). **C** The photo showed that the incision was well healed, with no swelling and thrill (arrow). **D** Doppler ultrasound showed the radial artery remained intact, with antegrade flow preserved

Intraoperatively, the artery and the vein within the fistula were mixed up and hard to identify. Dissecting according to the proximal radial artery course till the bump of the fistula, the vessel clamp was deployed (Fig. 2A). After ligating the fistula and resecting 5 mm of the radial artery, the anastomosis of the radial artery was reconfirmed and sutured with 8-0 prolenen under the microscope (Fig. 2B). Closure of the incision was performed after checking that the radial artery had a smooth blood flow, as well as there was no bleeding from the anastomosis.

In almost a week following the surgery, the numbness has gradually improved and the surgical incision was well healed. On physical examination, there was no difference in radial pulses on both sides. At the 20-month follow-up, the physical examination showed no recurrence of swelling or palpable thrill at the surgical site (Fig. 2C). Both static two-point discrimination and Semmes–Weinstein monofilament results showed a significant improvement at the middle finger (5 mm and 3.61, respectively). Two-point pinch force increased from 2.5 kg preoperatively to 6 kg at the last follow-up. A repeat ultrasound showed the radial artery remained intact, with antegrade flow preserved (Fig. 2D).

## Discussion and conclusions

In recent years, the radial access for cardiac catheterization is now increasingly preferred rather than femoral approach due to its advantages, which contain comparable effectiveness, less bleeding, and lower number of complications [5]. In patients with acute coronary syndromes (ACS), these benefits are more evident [6]. Nevertheless, Multiple sequelae encountered with TRA such as radial artery occlusion, hematomas, pseudoaneurysm and AVF, although less frequent, do occur [7]. The characteristics of previous radial AVF cases relative to catheter-based procedures have been reviewed [1, 3, 5, 8–21]. The clinical manifestations of AVFs are mainly related to venous dilation as the arterial blood bypasses the tissues and capillaries, returning directly to veins [8]. However, neurological complication secondary to radial AVFs is extremely rare. In this case, we presented a patient diagnosed with ischemic steal syndrome related to radial AVF.

Steal syndrome was diagnosed based on decreased or absent distal pulse, coolness, pain, abnormal skin color, ischemic ulceration of digits, numbness, sensory impairment, or motor impairment. Access-induced hand ischemia is a relatively rare but dreaded complication in patients with fistulas [22]. In addition, vascular steal syndrome needs to be distinguished from peripheral neuropathy and carpal tunnel syndrome (CTS), which can also cause pain and/or numbness of the hands [23]. Bauer et al. reported a case of carpal tunnel syndrome presented with numbness and paresthesia in the radial 1–3 fingers after percutaneous coronary intervention [24]. Electroneurography showed that the median nerve motor latencies at the wrists were prolonged, and the sensory nerve velocity and amplitude were reduced. The AVF has been identified as a risk factor for developing CTS in hemodialysis patients. In this case, considering the Tinel’s sign and Phalen’s test was negative, the image of CT angiography and the numbness gradually improved in a short time, we were more likely to diagnose the steal syndrome. In addition, this patient presented with pain and numbness in the fingers but did not progress to gangrene 11 months after the fistula formation, which may be due to the low shunts. Upon confirming the diagnosis, surgery was immediately undertaken to avoid further deterioration.

The purpose of AVF treatment is to close the fistula while maintaining the necessary blood supply. Due to the scarcity of cases, there has been no clear recommendation for the treatment of radial AVF. In Table 1, we reviewed the first-treatment strategies used in 17 cases of radial AVF: 6 of these patients were treated conservatively, 10 underwent surgery, and 1 received endovascular treatment.

**Table 1:** Cases that developed arteriovenous fistula associated with radial catheter-based procedures

Studies	Age/Sex	Symptoms	Time to presentation	Diagnostic tool	Treatments	Outcomes
Dutton et al. [1]	61/F	1. Pain 2. Paresthesias and thrill	2 months later	Doppler ultrasound	Surgery	Good
Shah et al. [3]	71/M	1. Dilated veins 2. Palpable thrill	1 week later	Doppler ultrasound	Compression	Good
De Oliveira et al. [5]	86/M	1. Swelling 2. Palpable thrill	1 year later	Doppler ultrasound	Surgery	Good
Kwac et al. [8]	67/M	1. Swelling 2. Palpable thrill	1 year later	Doppler ultrasound CT angiography	Surgery	Good
Na et al. [9]	61/F	1. Palpable thrill 2. Dilated veins	11 months later	Doppler ultrasound CT angiography	Compression	Poor
Nagata et al. [10]	68/M	1. Pulsatile mass	7 years later	CT angiography	Surgery	Good
Pulikal et al. [11]	64/M	1. Dilated veins 2. Palpable thrill	5 weeks later	Doppler ultrasound	Surgery	Good
Yang et al. [12]	54/M	1. Palpable thrill	2 months later	Doppler ultrasound	Surgery	Good
Goldberg et al. [13]	55/F	1. Dilated veins 2. Palpable thrill	2 months later	Doppler ultrasound	Surgery	Good
Dehghani et al. [14]	62/M	1. Palpable thrill	1 month later	Doppler ultrasound	Compression	Good
Spence et al. [15]	61/M	1. Swelling 2. Palpable thrill	1 year later	Doppler ultrasound	Surgery	Good
Summaria et al. [16]	66/M	1. Swelling 2. Pain, thrill	1 year later	Doppler ultrasound	Compression	Poor
A et al. [17]	45/M	1. Swelling 2. Pain	1 month later	CT angiography	Surgery	Good
Herzallah et al. [18]	85/M	1. Palpable thrill 2. Pain	2 months later	Doppler ultrasound	Surgery	Good
Hashimoto et al. [19]	61/M	1. Painful swelling 2. Bruit	1 week later	Doppler ultrasound	Compression	Good
Sugahara et al. [20]	74/F	1. Swelling 2. Murmur and thrill	8 months later	Doppler ultrasound CT angiography	Endovascular	Good
Regueiro et al. [21]	55/M	1. Painful swelling 2. Thrill	9 months later	Doppler ultrasound	Compression	Poor

Unlike the femoral artery, the shallow location and small lumen make the radial artery easy to compress, so ultrasound-guided compression may be enough to prevent complications of AVFs that can be avoided [2]. In the previous cases, nearly half of the compression therapy had failed, and surgery or endovascular treatment was eventually employed. In particular, the successful cases were treated at the early stage of the disease (about 1 month), while failed cases were diagnosed late (almost 1 year) [3, 9, 14, 16, 19, 21]. Taking this into account, we believed that compression therapy could work as intended, but care must be taken of the adaptive time.

Surgical treatment has been advocated in those with obvious paresthesias and additional complications. In most previous cases, patients with AVF were treated with surgery, which was an invasive procedure but had a definite effect. There are several options for surgical treatment depending on the size and location of an AVF, including ligation, excision, and repair [5, 10, 18]. Most fistulas, mainly because they were easy to identify, were frequently treated with ligation. However, when the fistula tissue was mixed and difficult to distinguish, or the radial artery was severely damaged and even hemodynamics was affected, further repair of the artery was required [1].

**Fig. 3:**
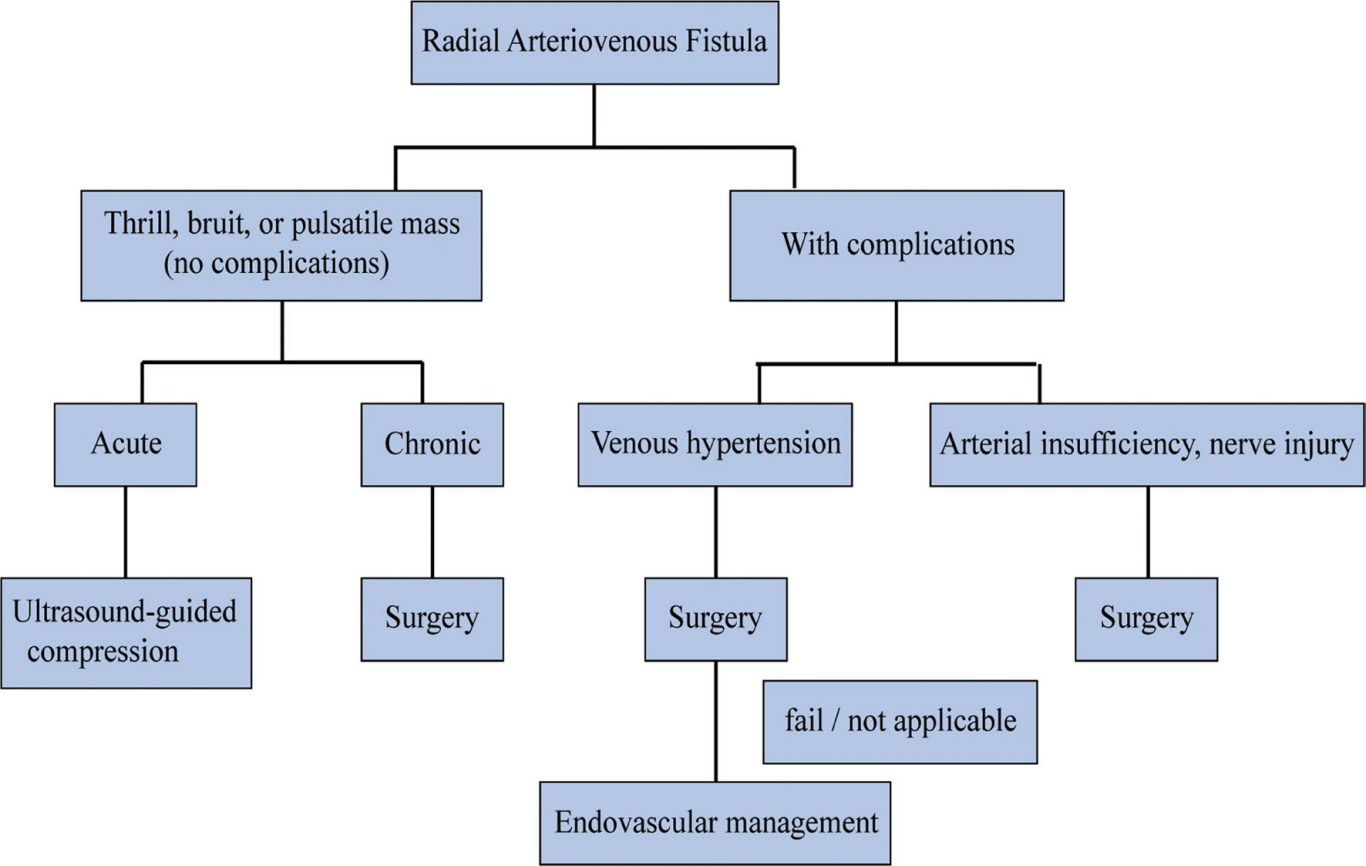
Treatment algorithm for the management of radial Arteriovenous Fistula

Due to inherent limitations such as the small size of the artery and embolism risk, endovascular treatment has not been used before. Summaria et al. performed the first percutaneous closure of a fistula after the failure of ultrasound-guided compression therapy [16]. Moreover, Sugahara et al. reported a method of balloon-assisted percutaneous embolization performed on an AVF patient who refused surgical ligation [20]. In short, while the vascular interventional procedure is attractive, it is complicated and expensive and should only be performed when the surgery is not indicated. In light of the above evidence, we proposed a treatment strategy for radial AVFs, as shown in Fig. 3.

In summary, this case suggests that ischemic steal syndrome can be complicated with radial AVF after cardiac catheterization. With the rapid development of transradial cardiac catheterizations, related complications such as AVFs, although rare, have gradually been discovered. We believe that once an AVF is diagnosed, early treatment options such as compression or surgery are necessary to relieve symptoms and prevent further complications.

## Data Availability

All data generated or analyzed during this manuscript are included in this published article.

## Abbreviations

AVF: Radial arteriovenous fistula
CTA: Computed tomography angiography
CTS: Carpal tunnel syndrome
